# The obesity paradox in intracerebral hemorrhage: a systematic review and meta-analysis

**DOI:** 10.3389/fendo.2023.1255538

**Published:** 2023-11-29

**Authors:** Zexu Wang, Jie Wang, Jiayan Wang, Yinghua Liao, Xin Hu, Manni Wang

**Affiliations:** ^1^Department of Neurosurgery, West China Hospital, Sichuan University, Chengdu, Sichuan, China; ^2^Department of Hematology, West China Hospital, Sichuan University, Chengdu, Sichuan, China; ^3^Department of Biotherapy, Cancer Center, West China Hospital, Sichuan University, Chengdu, Sichuan, China

**Keywords:** obesity, intracerebral hemorrhage, obesity paradox, mortality, stroke

## Abstract

**Background:**

Intracerebral hemorrhage (ICH) has a mortality rate which can reach 30–40%. Compared with other diseases, obesity is often associated with lower mortality; this is referred to as the ‘obesity paradox’. Herein, we aimed to summarize the studies of the relations between obesity and mortality after ICH.

**Method:**

For this systematic review and meta-analysis (PROSPERO registry CRD42023426835), we conducted searches for relevant articles in both PubMed and Embase. Non-English language literature, irrelevant literature, and non-human trials were excluded. All included publications were then qualitatively described and summarized. Articles for which quantitative analyses were possible were evaluated using Cochrane’s Review Manager.

**Results:**

Ten studies were included. Qualitative analysis revealed that each of the 10 studies showed varying degrees of a protective effect of obesity, which was statistically significant in 8 of them. Six studies were included in the quantitative meta-analysis, which showed that obesity was significantly associated with lower short-term (0.69 [0.67, 0.73], p<0.00001) and long-term (0.62 [0.53, 0.73], p<0.00001) mortality. (Data identified as (OR [95%CI], p)).

**Conclusion:**

Obesity is likely associated with lower post-ICH mortality, reflecting the obesity paradox in this disease. These findings support the need for large-scale trials using standardized obesity classification methods.

**Systematic review registration:**

https://www.crd.york.ac.uk/prospero/display_record.php?ID=CRD42023426835, identifier CRD42023426835.

## 1 Introduction

Intracerebral hemorrhage (ICH), a nontraumatic hemorrhage caused by blood vessel ruptures in the brain parenchyma, accounts for 20–30% of all strokes and 30–40% of deaths during the acute phase (1). ICH has an overall incidence of 24.6 per 100.000 patient years, and is higher in Asian populations (2).

Obesity is a well-established risk factor for increased morbidity and mortality among the general population (3, 4). According to previous studies, obesity is a strong predictor of a number of diseases, including diabetes, cardiovascular disease, hypertension, etc. (5, 6) However, a theoretical ‘obesity paradox’ indicates that obese patients may have a better outcome than non-obese patients among patients with the same disease. The obesity paradox was first identified in those with cardiovascular disease (7, 8). In a multivariate analysis of 1203 patients with advanced heart failure, high BMI was a strong predictor of good prognosis (7). Research on the obesity paradox can guide us in the nutritional management of our patients and may have a positive effect on improved outcomes. With the global increase in obesity (9, 10) and the discovery of an obesity paradox in a variety of diseases (11), the importance of in-depth study of this phenomenon has become increasingly important.

Past studies of the relations between stroke and obesity have demonstrated that the latter is independently associated with favorable functional recovery at 3-month follow-up (12). Although several ICH studies exist (13–20), their results have been contradictory. Some have found significant protective effects, while others demonstrated an association with higher mortality (14). Therefore, a summary and analysis of past study results on the relations between outcomes after ICH and obesity is warranted.

Herein, we aimed to systematically review the literature addressing a possible obesity paradox in patients with ICH, and to combine currently available data using meta-analysis to estimate the role of the obesity in ICH.

## 2 Methods

### 2.1 Study design

This systematic review and meta-analysis were conducted and registered according to PROSPERO (CRD42023426835).

### 2.2 Search strategy and process

Briefly, the search was based on both keywords and index terms using two databases: PubMed and Embase. The summary search strategy was “cerebral hemorrhage” and “BMI/obesity”. The first screening was carried out independently by two reviewers, each based on the title and abstract, with any disagreements resolved by a third reviewer. The second screening was done by reading the full texts in consultation between the two reviewers.

### 2.3 Study selection and data extraction

We included articles that contained data related to BMI, data related to ICH outcomes. Exclusion criteria include: 1. Case reports, case series, letters, commentaries, book chapters, animal studies, and descriptive studies without calculated risk estimates (hazard ratio [HR], odds ratio [OR], or relative risk); 2. Non-English articles; 3. Studies without statistical calculations and data analyses; 4. Studies without separate data related to patients with ICH, including those that classified ICH as stroke for statistical purposes.

The first author, country, year of article publication, total number of patients with ICH, gender ratio, mean age, follow-up time, obesity classification criteria, and baseline outcome indicators were extracted.

### 2.4 Quality assessment

The included literature was evaluated using the Newcastle-Ottawa Scale (NOS). Comparability on the most important factors was defined (i.e., hypertension, hyperlipidemia, hyperglycemia). A median follow-up time >1 month was considered sufficiently long. Missed follow-up at rates <20% were considered adequate. One point was added if the evaluation indicator was explicitly stated in the text. No points were added if they were not explicitly stated or could be inferred as absent. A total score >8 was considered to indicate a high quality study.

### 2.5 Obesity definition

The body mass index (BMI) is an internationally applied tool used to screen weight for height and serves as a health indicator. The BMI formula = (weight ÷ height^2^), where weight is in kilograms and height is in meters. Due to differences in BMI classifications, we standardized the definition of obesity in quantitative analyses herein.

In addition to obese versus nonobese dichotomization, we also adopted BMI as a more detailed obesity classification criterion. For Eastern populations, using the WHO BMI classification, we redefined ‘overweight’ or ‘obese’ as obese and ‘underweight’ and ‘normal weight’ as nonobese. For Western populations, BMI >30 was considered obese. If no specific classification was mentioned in the article, BMI was disregarded and the authors’ obesity classification was used.

### 2.6 Statistical analysis

We categorized the studies included in the meta-analysis into long- and short-term mortality comparisons according to their follow-up times. Short-term mortality was defined as in-hospital mortality and 30-day mortality; later mortality was defined as long-term mortality.

Statistical analyses were performed using pooled OR with 95% confidence intervals (95%CI), and dichotomous variables were calculated using the Mantel-Haenszel method and fixed/random effects models. The Mantel-Haenszel model for determining linear relationships between ordered categorical and dichotomous variables is applicable to this study. Heterogeneity of the included studies was tested using the Cochrane I2 test, with a threshold of p<0.10 indicating the presence of heterogeneity. If there was no heterogeneity, a fixed-effects model was applied. Otherwise, a random-effects model was used. For analyses using the random effects model, if the combined term is less than 5 (including 5), we will use the Hartung-Knapp adjusted approach for correction (21, 22). A Z-test was performed for the overall effect, and p<0.05 was considered statistically significant. In this study, the Z-test was able to show that the mean mortality rate of obese ICH patients differed from that of non-obese ICH patients. A sensitivity analysis will be conducted to determine the stability of the findings using a literature-by-literature exclusion approach. Data analyses were conducted using Review Manager 5.4.1. and R 4.2.1.

## 3 Results

### 3.1 Search results and study characteristics

**Figure 1:**
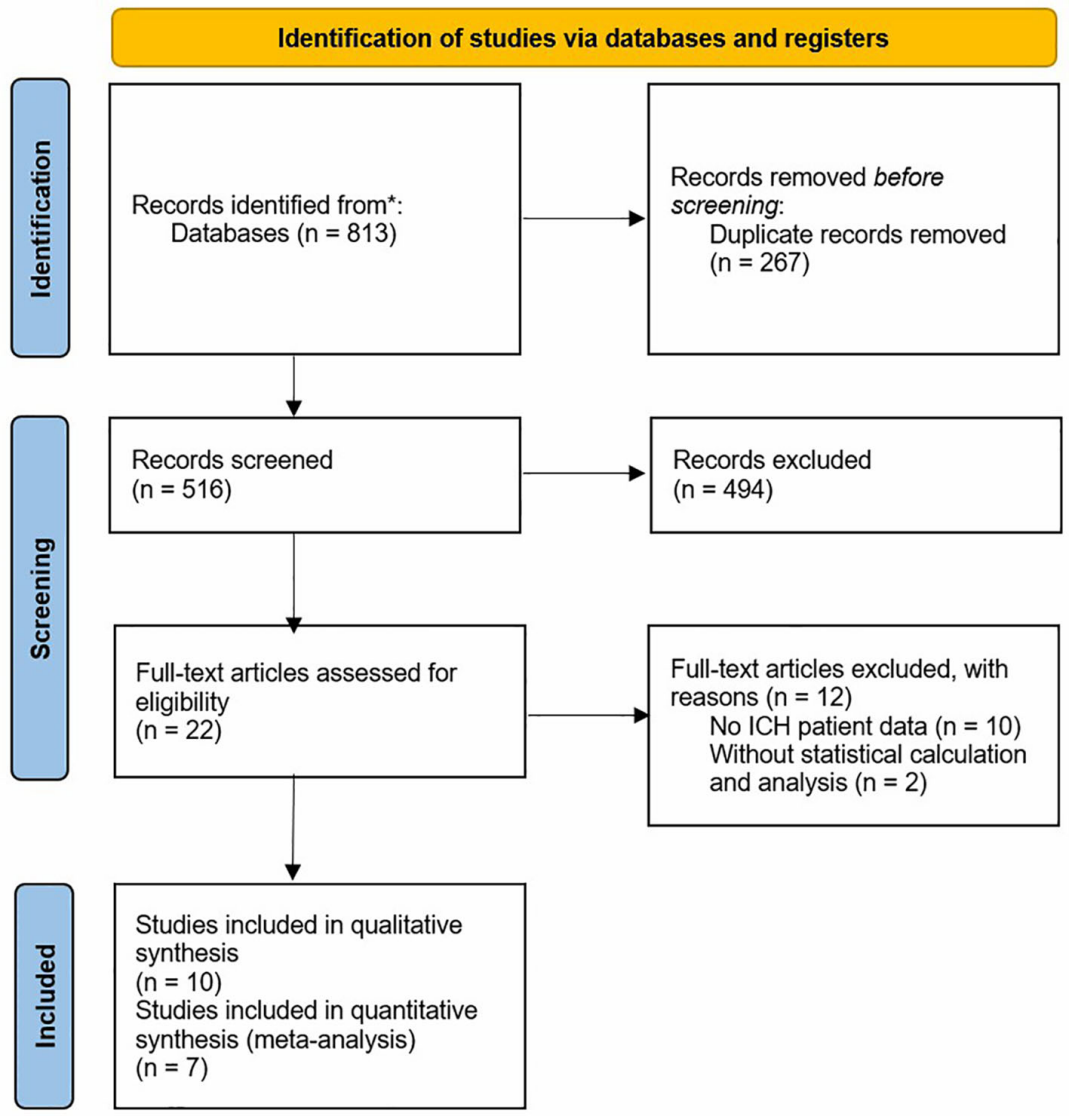
Flow diagram of study selection process.

The study selection process is summarized in **Figure 1**, constructed based on the PRISMA statement, and the research includes 10 studies in the end. A final ten studies, representing a cumulative 567,766 patients with ICH, were included.

Each of the eight included studies had a sample size >1,000 patients with ICH, which is considered relatively large. Follow-up durations of the included literature varied from in-hospital to over 10 years, with multiple follow-up periods present within some studies. In addition to mortality, other quantitative prognostic outcomes included the modified Rankin Scale (mRS), special disposition rate, and prolonged discharge rate. Of the eight included papers, two did not provide specific case data, so the final meta-analysis was performed on six papers.

Study characteristics varied considerably among studies (**Table 1**).

**Table 1:** Study characteristics.

First Author	Country	Year	ICH Cases	Mean Age	% male	Follow-up Time	Obesity Categories (kg/m^2^)	Outcomes
Western population								
V. Javalkar	US	2020	47,700	68	52	In-hospital	NR	In-hospital mortality: 24%
H. Hoffman	US	2020	123,415	NR	49.6	In-hospital	BMI 30–39.9 BMI ≥ 40	Overall mortality: 28.1% Non-routine discharge disposition: 81.1% Tracheostomy: 4.5% Gastrostomy: 8.5% Median LOS: 5.0d (interquartile range 2.0–9.0) Mean ( ± standard deviation) Inflation-adjusted hospital charge: $67,229 ± 106,479
H. Hoffman	US	2020	751	61 (median)	54.2	30-day	BMI 30–39.9 BMI ≥ 40	30d mortality: 23.3% Non-routine discharge: 85.1% eLOS: 21.2%
S. R. Persaud	US	2019	99,212	68.78	NR	In-hospital	BMI < 30 BMI 30–39.9 BMI ≥ 40	In-hospital Mortality: 25.2% Non-routine discharge disposition*: 74.9% Extended hospitalization: 37.8% Tracheostomy/gastrostomy: 10.0% VP shunt: 0.9%
N. S. Dangayach	US	2018	202	61	57	90-day	BMI < 25 BMI ≥ 25	90d mortality: 41% mRS score 4–6 (moderate–severe disability or death): 66%
Asian population								
W. Sun	China	2016	1,572	61.1	59.8	3-month 12-month	BMI <18.5 BMI 18.5–23 BMI 23–25 BMI ≥25	12m mortality: 26.2% 3m death or high dependency: 54.0% 12m death or high dependency: 51.4%
B. J. Kim	Republic of Korea	2011	1,356	59.7	55.2	30-day 33.6-month (mean)	BMI < 18.5 BMI 18.5–23.0 BMI 23.0–24.9 BMI 25.0–29.9 BMI > 30.0	30d mortality: 7.2% Long-term mortality: 26.9%
I. Oki	Japan	2006	9,526	50.6	43.8	17.3-year (mean)	BMI < 18.5 BMI 18.5–23.0 BMI 23.0–24.9 BMI 25.0–29.9 BMI > 30.1	Overall mortality: 0.04%
Z. Cao	China	2021	82,789	NR	62.4	In-hospital	BMI < 18.5	In-hospital mortality: 2.3% Non-routine disposition: 8.5%
							BMI 18.5–22.9	
							BMI 23.0–24.9	
							BMI > 25.0	
N. Yoshida	Japan	2022	201,243	NR	NR	In-hospital	BMI < 18.5	Overall population outcome NR
							BMI 18.5–23.0	
							BMI 23.0–24.9	
							BMI 25.0–30	
							BMI > 30	

BMI, body mass index; NR, not reported; LOS, length of stay; Elos, extended length of stay; US, United States; and VP, ventriculoperitoneal. *shown in the text as routine discharge disposition. #High dependence defined as mRS score 3–5.

#### 3.1.1 Study 1

In this Japanese study, Oki et al. (18) investigated the relations between BMI and stroke mortality (including cerebral infarction and ICH) among 9,526 men and women aged 30 years or older who were randomly selected throughout Japan in 1980. These participants were followed for 19 years. HR and 95%CI were examined by Cox’s proportional hazards regression models, including BMI levels.

#### 3.1.2 Study 2

To examine the association between BMI and functional outcome, Dangayach et al. (13) analyzed 202 patients admitted to the neurological intensive care unit (ICU) who were prospectively enrolled in the Columbia University ICH Outcomes Project. Patients were divided into two groups: overweight (BMI ≥25 kg/m^2^) and not overweight (BMI <25 kg/m^2^). BMI was calculated using data collected at the time of the initial emergency department or in-hospital evaluation. Exact mortality was not mentioned.

#### 3.1.3 Study 3

Javalkar et al. (16) analyzed 47,700 patients with ICH in the United States (US) National Inpatient Sample (NIS) database from 2012–2015. BMI classification standards were not reported.

#### 3.1.4 Study 4

Sun et al. (20) used ChinaQUEST, a hospital-based register, to analyze 1,571 patients with ICH. BMI categories were underweight (BMI <18.5 kg/m^2^), normal weight (BMI 18.5–23 kg/m^2^), overweight (BMI 23–25 kg/m^2^), and obese (BMI ≥25 kg/m^2^).

#### 3.1.5 Study 5

In their study using the NIS, Persaud et al. (19) analyzed the 99,212 patients from 2007–2014. BMI categories were nonobese (BMI <30 kg/m^2^), obese (BMI ≥30 and ≤40 kg/m^2^), and morbid obese (BMI >40 kg/m^2^).

#### 3.1.6 Study 6

Hoffman et al. (15) analyzed data from 123,415 patients with ICH in the NIS from 2002–2011 to examine the effects of obesity on outcomes after spontaneous ICH. The BMI categories were the same used in study 5.

#### 3.1.7 Study 7

This South Korean study analyzed 1,604 patients with ICH. Kim et al. (17) analyzed the association between obesity and 30-day and long-term mortality risks.

#### 3.1.8 Study 8

Data for this study were from craniotomies for evacuation in supratentorial ICH from 2006–2017, and included 751 patients. Hoffman et al. (14) analyzed 30-day mortality, non-routine discharge disposition, and extended length of stay (eLOS, defined as the top quartile for the cohort).

#### 3.1.9 Study 9

Cao et al. (23) evaluated the associations between BMI and in-hospital mortality, complications, and discharge disposition in ICH using data derived from the China Stroke Center Alliance, a national, hospital-based, multicenter, voluntary, quality assessment and improvement initiative performed in China. A total of 82,789 patients were involved from August 2015–July 2019.

#### 3.1.10 Study 10

Yoshida et al. (24) analyzed 5,020,464 records from the national Japanese Registry of All Cardiac and Vascular Diseases-Diagnosis Procedure Combination dataset over from 2012–2019. They evaluated BMI trends and its impact on in-hospital mortality rates within six acute cardiovascular disease types: acute heart failure, acute myocardial infarction, acute aortic dissection, ischemic stroke (IS), ICH, and subarachnoid hemorrhage.

### 3.2 Risk-of-bias and quality of studies

Based on the NOS scale, we evaluated each article in three broad areas: selection, comparability, and outcome. The results of this evaluation are in **Table 2**. No study scored >7 points. It is worth noting that the included studies did better on both selection and outcomes, while scoring lower on comparability of both important and other factors, which may have led to higher heterogeneity in our meta-analysis.

**Table 2:** Quality evaluation.

Study	Selection				Comparability		Outcome			Score
	Representativeness	Selection of non-exposed	Ascertainment of non-exposed	No sudden death from ICH	Comparability on most important factors	Comparability on other risk factors	Assessment of outcome	Sufficient follow-up duration	Follow-up adequacy	
Oki et al.	√	√	√	√	N/A	N/A	√	√	√	7
Kim et al.	√	√	√	—	—	—	√	√	√	6
Sun et al.	√	√	√	√	—	—	√	√	√	7
Dangayach et al.	√	√	√	—	√	—	√	√	—	6
Persaud et al.	√	√	√	—	√	—	√	N/A	√	6
Hoffman et al.	√	√	√	√	√	—	√	√	√	5
Hoffman et al.	√	√	√	—	—	—	√	N/A	√	5
Javalkar et al.	√	√	√	—	—	—	√	N/A	√	5
Cao et al.	√	√	√	√	—	—	√	—	√	6
Yoshida et al	√	√	√	√	—	—	√	—	√	6

√ represents reported, N/A not applicable: unknown risk, —: unmentioned.

### 3.3 Qualitative synthesis

#### 3.3.1 Short-term mortality

As described above, we identified in-hospital and 30-d mortality as ‘short-term mortality’. Six studies analyzed in-hospital mortality (studies 3, 5, 6, 9, and 10) and two analyzed 30-day mortality (studies 7 and 8).

In studies 3 (OR 0.64, 95%CI 0.59–0.69), 5 (OR 0.69, 95%CI 0.62–0.76), and 6 (OR 0.62, 95%CI 0.56–0.69), an association was found between obesity and lower mortality, while the protective effect of morbidly obesity was weaker than that of obesity. In study 8, despite the trend toward a protective effect of obesity, statistical significance was not found (OR 0.624, 95%CI 0.381–1.022); interestingly, in their analysis of morbid obesity, excessive BMI (>40) was associated with higher mortality (OR 2.175, 95%CI 1.034–4.578). In study 9, underweight (OR 2.057, 95%CI 1.193–3.550) was associated with higher mortality rates. In study 10, overweight (OR 0.87, 95%CI 0.4–0.90) and obese I (OR 0.93, 95%CI 0.90–0.97) were protective, while underweight was associated with higher mortality (OR 1.26, 95%CI 1.22–1.31).

There were no statistically significant results reported in study 7.

#### 3.3.2 Long-term mortality

In study 1, compared with the reference group (BMI 25.0–29.9), the only protective effect was in the group with BMI 25.0–29.9 (HR 0.83, 95%CI 0.36–1.91). In the group with BMI ≥30.0, this became a harmful mortality factor. However, this was not a statistically significant effect.

Study 4 analyzed 12-month mortality with Cox proportional hazard models. Both the unadjusted HR and the adjusted HR (0.75, 95%CI 0.59–0.95 and HR 0.71, 95%CI 0.56–0.91, respectively) showed significant effects of obesity on lower mortality. In addition, the investigators analyzed for mortality or high dependence within 3- and 12-month follow-up. All results showed a protective effect of obesity, to varying degrees.

In study 7, overweight (HR 0.69, 95%CI 0.49–0.96) and obese (HR 0.61, 95%CI 0.43–0.88) were protective factors. Underweight was also significantly associated with higher mortality (HR 1.64, 95%CI 1.11–2.40).

In addition, Dangayach use the mRS score to evaluate ICH outcomes (13), dividing patients into those with an mRS score 0–3 or mRS score 4–6. Higher BMI (BMI ≥25 kg/m^2^) was associated with better outcomes (adjusted OR 2.05, 95%CI 1.03–4.06, p=0.04). In-hospital mortality data were also collected.

**Table 3** shows mortality risk findings for each study.

**Table 3:** ICH studies reporting associations between BMI and mortality.

First author	Follow-up	Analyses by sex	BMI categories (kg/m^2^)	Risk of mortality (OR(95%CI))	
				Univariate analysis	Multivariate analysis
Western population					
V. Javalkar	In-hospital	—	NR	—	0.64 (0.59–0.69)
H. Hoffman	In-hospital	—	Nonobese (<30)	—	REF
		—	Obese (30 to <40)	—	0.62 (0.56–0.69)
		—	Morbidly obese (≥40)	—	0.76 (0.66–0.88)
H. Hoffman	30-day	—	Nonobese (<30)	—	REF
		—	Obese (30 to <40)	—	0.624(0.381 – 1.022)
		—	Morbidly obese (≥40)	—	2.175(1.034 – 4.578)
S. R. Persaud	In-hospital	—	Nonobese (<30)	REF	REF
		—	Obese (30 to <40)	0.60 (0.55–0.65)	0.69 (0.62–0.76)
		—	Morbidly obese (≥40)	0.77 (0.70–0.86)	0.85 (0.74–0.97)
N. S. Dangayach	90-day	—	<25	REF	—
		—	≥25	0.70 (0.39, 1.26)	—
Asian population					
W. Sun	12-month	—	Underweight (<18.5)	0.97 (0.67–1.41)	0.78 (0.54–1.15)
		—	Normal weight (18.5 to <24)	REF	REF
		—	Overweight (24 to <28)	0.8 (0.62–1.03)	0.83 (0.63–1.08)
		—	Obese (≥28)	0.75 (0.59–0.95)	0.71 (0.56–0.91)
B. J. Kim	30-day	—	Underweight (<18.5)	—	0.75 (0.26–2.11)
		—	Normal weight (18.5 to <23)	—	REF
		—	Overweight (23 to <25)	—	0.86 (0.47–1.56)
		—	Obese (≥25)	—	0.89 (0.47–1.68)
	Long-term	—	Underweight (<18.5)	—	1.64 (1.11–2.40)
		—	Normal weight (18.5 to <23)	—	REF
		—	Overweight (23 to <25)	—	0.69 (0.49–0.96)
		—	Obese (≥25)	—	0.61 (0.43–0.88)
I. Oki	17.3-year	Both	<18.5	—	1.23 (0.46, 3.29)
			18.5 to <23.0	—	1.26 (0.67, 2.37)
			23.0 to <25.0	—	REF
			25.0 to <30.0	—	0.83 (0.36, 1.91)
			≥30.0	—	2.31 (0.65, 8.26)
		Men	<18.5	—	1.00 (0.24, 4.12)
			18.5 to <23.0	—	1.37 (0.55, 3.40)
			23.0 to <25.0	—	REF
			25.0 to <30.0	—	1.36 (0.44, 4.23)
			≥30.0	—	6.61 (0.79, 55.65)
		Women	<18.5	—	1.55 (0.39, 6.24)
			18.5 to <23.0	—	1.19 (0.48, 2.95)
			23.0 to <25.0	—	REF
			25.0 to <30.0	—	0.50 (0.15, 1.72)
			≥30.0	—	1.47 (0.30, 7.25)
Z. Cao	In-hospital	—	Underweight (<18.5)	1.493 (1.216–1.833)	2.057 (1.193–3.550)
		—	Normal weight (18.5 to <23)	REF	Reference
		—	Overweight (23 to <25)	0.861 (0.768–0.966)	0.887 (0.647–1.216)
		—	Obese (≥25)	1.032 (0.926–1.151)	1.141 (0.846–1.539)
N. Yoshida	In-hospital	—	Underweight (<18.5)	—	1.26 (1.22–1.31)
		—	Normal weight (18.5 to <23)	—	REF
		—	Overweight (23 to <25)	—	0.87 (0.40–0.90)
		—	Obese I (25.0 to <30)	—	0.93 (0.90–0.97)
		—	Obese II (>30)	—	1.00 (0.94–1.07)

NR, not reported; REF, reference.

### 3.4 Meta-analysis

**Figure 2:**
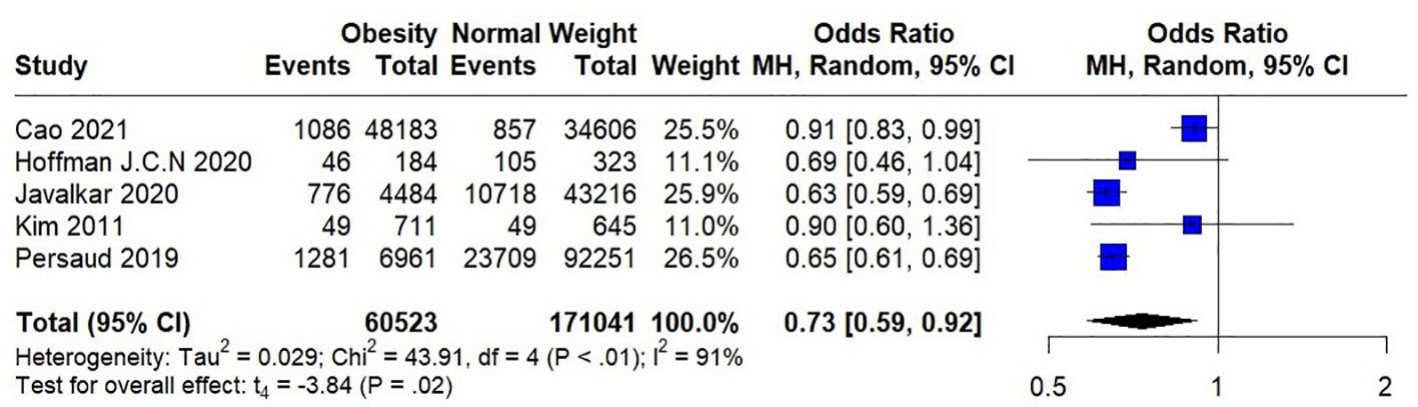
Forest plot of effect of obesity versus normal weight on short-term mortality.

**Figure 3:**
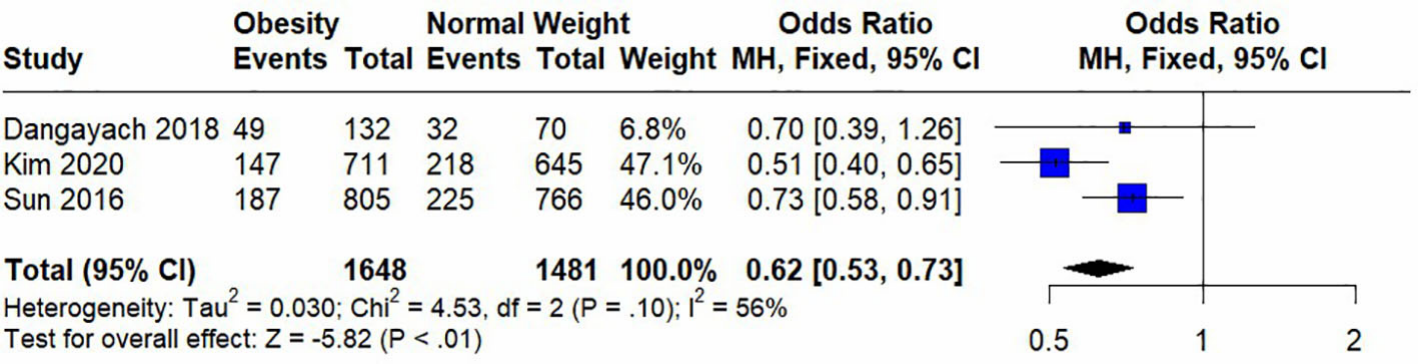
Forest plot of effect of obesity versus normal weight on long-term mortality.

Seven studies were included in the meta-analysis, among which four included short-term mortality data and three reported long-term mortality data. Their quantitative results are expressed as (OR [95%CI], p). In the analysis of heterogeneity, we found heterogeneity in short-term mortality data, (p<0.00001) and used a random-effects model. Long-term mortality data were not heterogeneous, (p=0.01) and fixed-effects models were used. These analyses are presented in two forest plots (**Figures 2**, **3**).

In the pooled analysis, obesity was associated with significantly lower short-term (0.73 [0.62, 0.88], p=0.0006) and long-term (0.62 [0.53, 0.73], p<0.00001) mortalities. Due to the small number of studies included in the analysis of short-term mortality and the use of a random-effects model, we calculated the results using the Hartung-Knapp adjusted approach. The result obtained was 0.73 [0.59; 0.91], p=0.0183, indicating that obesity is still significantly associated with lower short-term mortality.

**Figure 4:**
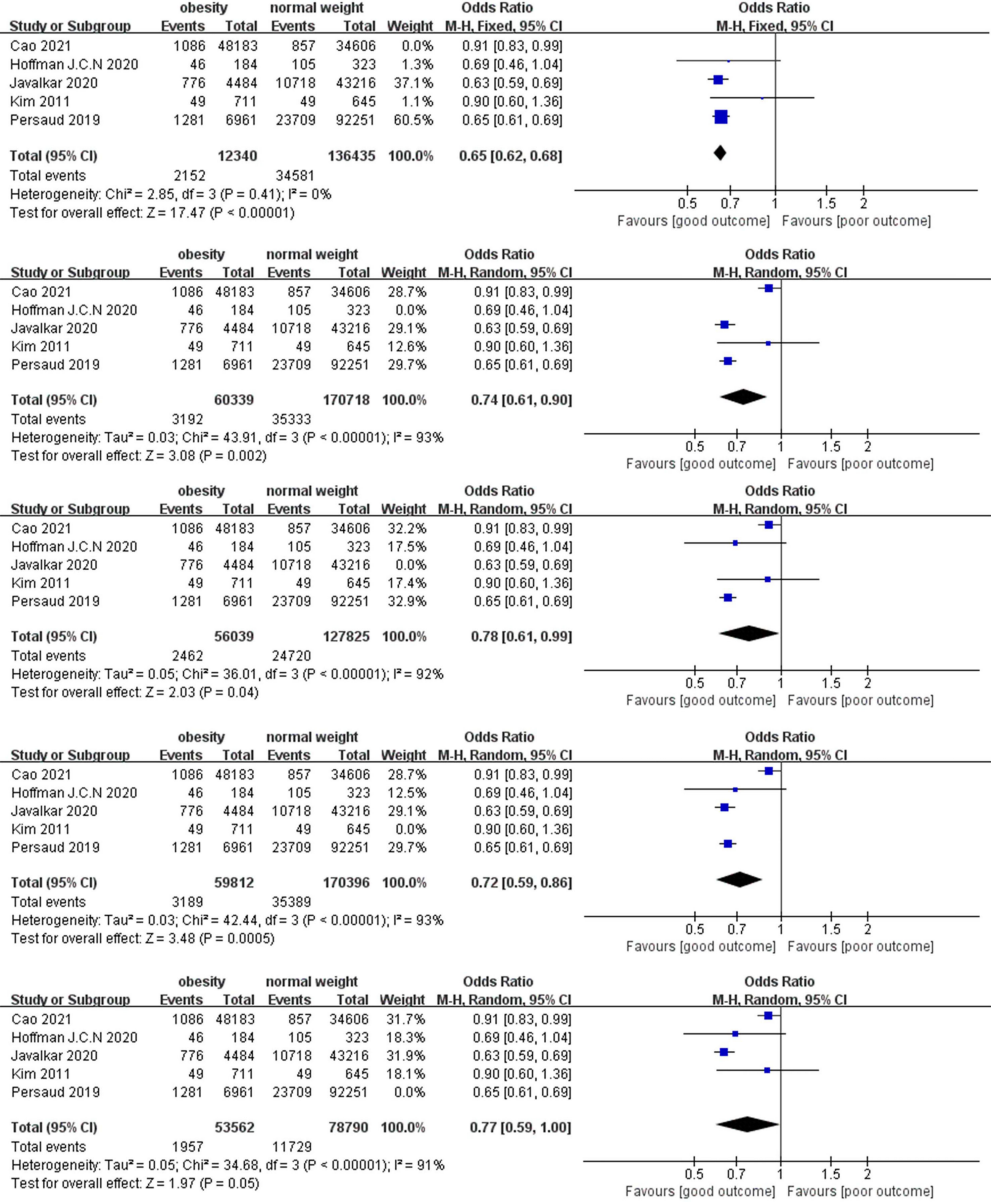
Sensitive analysis on short-term mortality.

**Figure 5:**
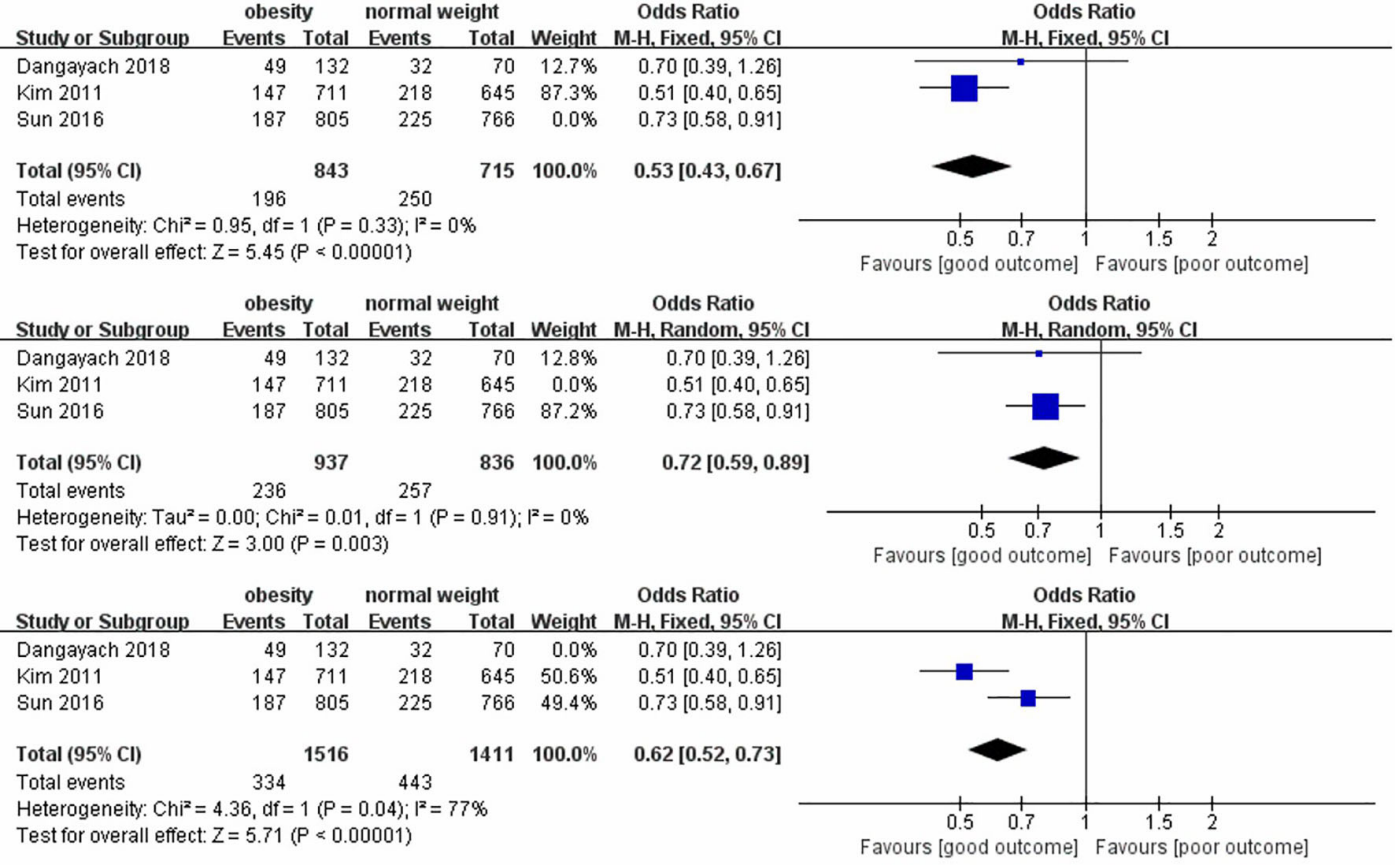
Sensitive analysis on long-term mortality.

In the sensitivity analysis, we excluded the included studies one by one and combined the remaining studies. The finding that obesity was associated with lower mortality remained unchanged in both the short-term and long-term mortality analyses, demonstrating the stability of the conclusions from the Meta-analysis. (**Figures 4**, **5**). Meanwhile, we found that heterogeneity in the analysis of short-term mortality was mainly caused by Study 9, which is consistent with the conjecture in the quality evaluation. Because of the stability of the results, we decided to keep this article in the quantitative analysis.

## 4 Discussion

All the studies included herein showed a protective tendency of obesity, or higher BMI, among patients with ICH against mortality, and with statistically significant findings (13, 16, 17, 19, 20). In our meta-analysis, statistically significant results were found.

Though the protective effect of obesity in ICH can be explained by physical mechanisms, this cannot be fully examined in the included studies due to methodological shortcomings. Patients with obesity have an increased metabolic reserve of adipose or muscle mass, which may help patients tolerate inflammatory events during treatment (17, 25). In addition to mortality, one study (13) also used the mRS to analyze the relations between obesity and outcome among patients with ICH, showing positive associations. Another potential mechanism of the obesity paradox is the resistance to unintentional weight loss from ICH, though weight loss is more likely in hemorrhagic stroke than in IS. In addition, unintentional weight loss at 3-month follow-up may be a more clinically relevant outcome (26).

Simultaneously, previous studies question the protective effect of morbid obesity (using the Western BMI categories). Several studies have shown a weakened protective effect in this case (15, 19) and, though not statistically significant, morbid obesity has been related to higher mortality (14). The reason for this inconsistency in the obesity paradox may be the higher complication rates with morbid obesity. For example, there is a relation between obesity and insulin resistance (27), and diabetes mellitus may contribute to poorer outcomes in ICH (28, 29).

In addition to the obesity findings, an effect of underweight was also noted in several studies. Some researchers contend that underweight is associated with worst outcomes from ischemic and hemorrhagic stroke (17, 30, 31). Yet when Sun et al. assessed these previous results, they found methodological inadequacies that called these conclusions into question (20).

This review was limited by the inclusion of too few articles, emphasizing the need for broader analyses. BMI categorization discrepancies also pose challenges to quantitative reviews. Herein, this problem was solved by using dichotomy variation, meaning that all data from patients with obesity were pooled despite BMI definition differences. In addition, whether BMI is a proper way to examine obesity among patients with ICH is unknown. Some authors have suggested that the interaction between cardiorespiratory fitness and BMI may play a more important role in understanding the obesity paradox (25). Ethnic differences in ICH mortality are also noteworthy. Sun W et al. noted that mortality among their cohort was obviously lower than that previously reported in Western patients with ICH (32). Thus, these results should be generalized to specific ethnic groups with great caution.

## 5 Conclusion

Quantitative meta-analysis showed that obesity is associated with lower post-ICH mortality. Qualitative analysis of each included article indicated similar trends toward the emergence of an obesity paradox, although more definitive statistical results are needed. Future research should assess the relations between morbid obesity and outcomes, include more standardized, empirical obesity measurements, and report data collection procedures more clearly. Such measures are encouraged, in part, due to the increasing numbers of patients with obesity.

## Data availability statement

The original contributions presented in the study are included in the article/supplementary materials. Further inquiries can be directed to the corresponding authors.

## Author contributions

ZW: Conceptualization, Data curation, Investigation, Methodology, Software, Visualization, Writing – original draft, Writing – review and editing. JieW: Conceptualization, Data curation, Formal Analysis, Investigation, Methodology, Software, Writing – original draft, Writing – review and editing. JiaW: Conceptualization, Investigation, Methodology, Software, Writing – original draft. YL: Conceptualization, Investigation, Methodology, Supervision, Writing – review and editing. XH: Conceptualization, Investigation, Writing – review and editing, Methodology. MW: Conceptualization, Investigation, Writing – review and editing, Data curation.
